# Transition Services for Children and Young Adults with Movement Disorders: A Survey by the MDS Task Force on Pediatrics

**DOI:** 10.1002/mdc3.13549

**Published:** 2022-09-28

**Authors:** Amit Batla, Jean‐Pierre Lin, Jitendra K. Sahu, Jonathan W. Mink, Tamara Pringsheim, Emmanuel Roze, Manju Kurian, Victor Fung

**Affiliations:** ^1^ Department of Clinical and Movement Neuroscience UCL Queen Square Institute of Neurology London United Kingdom; ^2^ Evelina Children's Hospital Guy's and St Thomas' NHS Foundation Trust London United Kingdom; ^3^ Advanced Pediatric Center Postgraduate Institute of Medical Education and Research Chandigarh India; ^4^ Department of Neurology University of Rochester Rochester New York USA; ^5^ Department of Clinical Neurosciences University of Calgary Calgary Alberta Canada; ^6^ Department of Neurology, Salpêtrière Hospital Sorbonne University and Assistance Publique ‐ Hôpitaux de Paris Paris France; ^7^ UCL Institute of Child Health London United Kingdom; ^8^ Movement Disorders Unit, Department of Neurology Westmead Hospital Sydney New South Wales Australia; ^9^ Sydney Medical School University of Sydney Sydney New South Wales Australia

**Keywords:** transition, pediatric movement disorders, childhood onset movement disorder

## Abstract

**Background:**

There is currently very limited data related to transition services for movement disorders.

**Objectives:**

Movement Disorders Society (MDS) Task Force on Pediatrics conducted a survey of current provision of transition for young adults with movement disorders.

**Methods:**

The survey questionnaire was based on review of available evidence, with questions designed to capture service location, transition clinic structure, and core issues discussed. The questionnaire was digitalized as an online survey and sent to all members of the MDS.

**Results:**

Responses were received from a total of 252 MDS members representing 67 countries. Of the responders, 59% confirmed that they provided transition clinics for adolescents with movement disorders. Overall, there was some consensus regarding transition services in terms of patient age at transition, movement disorder etiologies, staffing the service, and medical/social issues discussed.

**Conclusion:**

This survey provides first‐hand data of existing movement disorder transition services and provides useful insights on transition clinics.

Children with movement disorders form a heterogeneous patient group with symptoms presenting in a challenging developmental context.^1^ The diversity of clinical presentation, variability of adult neurology expertise in childhood‐onset movement disorders and the general paucity of evidence for most of the treatment modalities in this age group can complicate ongoing care.^1^ Transition services are mandated by many national guidelines or consensus statements.^2^, ^3^

Many medical conditions requiring continued care into adulthood already have established models of transition.^3^, ^4^ Despite the guidance and recommendations available, there remains a paucity of evidence for successful transition models and services.^4^, ^5^, ^6^, ^7^, ^8^, ^9^

The Movement Disorders Society (MDS) Task Force on Pediatrics conducted a survey on existing practices with an aim to understand the current structure and role of transition clinics for childhood‐onset movement disorders and to understand the models of transition care relevant to these.

## Methods

Members of the MDS pediatric task force developed a questionnaire for distribution to all MDS members. Task force members identified key transition issues through a review of available information^3^, ^4^, ^5^, ^6^, ^7^, ^8^ and task force group discussions. The questionnaire, consisting of 16 questions (Appendix S1), was drafted, and included input from the subgroup of four child and two adult neurologists that undertook the scoping review.^7^ The final questionnaire was subsequently approved by all members of the task force. Please refer to Appendix S2 for details of the survey and analysis.

## Results

The survey was sent electronically to the global MDS member email list and is expected to have reached inboxes of 11,601 members. The responses were received from a total of 252 members of MDS from 67 countries. Of these, 138 (55%) responders were adult neurologists, 55 (22%) were child (pediatric) neurologists, other responders worked in internal medicine, pediatrics, or other related specialties like neurorehabilitation.

### Geographic location

A map of countries of practice of the respondents is shown in Figure S1.

### Transition services

Of the responders, 148 (58.7%) confirmed provision of transitional clinics. The overall survey results are presented in Table 1 and detailed results follow here.

**TABLE 1:** Summary of survey results as percent of positive responses to each choice

Question	Response options	% of responses in favor
By what age is the transition from pediatric side to adult side complete in all patients attending your service	Less than 16	18.91
	16 to 17	27.70
	17 to 18	27.70
	18 to 19	14.86
	19 to 21	10.81
	19 to 21	0.00
	Over 21	3.37
	Other	20.27
In addition to a pediatric consultant/doctor, who all are present in the transition clinic at your service? (select all that apply)	Adult neurologist	71.62
	Physiotherapist	52.70
	Speech therapists	32.43
	Occupational therapists	29.72
	Social services representatives	22.97
	Specialist nurses	21.62
	Pediatric nurses	20.94
	Learning disability advisors	9.48
	Other (please specify)	24.14
What issues are discussed in the transition service? (select all that apply)	Clinical presentation, natural history, and previous and current management	69.59
	The young person's personal knowledge of their disease	68.91
	Disease related co‐morbidities (such as orthopedic, gastrointestinal, or neuropsychiatric issues, risk of mortality)	64.19
	General health issues (like BMI, nutrition, participation in physical activity) and well being	60.81
	The young person's personal knowledge of their medications and perceived stigmatization	58.10
	Career choices and further education	48.65
	Changes in family dynamics with increasing patient age	39.19
	Effect of puberty on disease and medication	36.48
	Sexuality, pregnancy, and reproductive issues	31.08
	Addiction, drugs, alcohol, and smoking	26.35
	Driving	21.62
	Other (please specify)	18.91
How is the clinical data transferred from pediatric to transitional/adult services? (select all that apply)	Transfer of clinical records	62.15
	Transfer of neuroimaging	33.64
	Standardized pro formas	24.30
	Transfer of multidisciplinary team reports	30.37
	Other (please specify)	17.76
What other ways or options are available to you to allow safe transition from childhood to adulthood for your patients with movement disorders? (select all that apply)	Referral services to adult clinics within your hospital/service	62.23
	Transfer of clinical records and/or neuroimaging through a standardized care pathway	42.92
	Transfer of clinical records and/or neuroimaging through GP	18.88
	Transfer of clinical records and/or neuroimaging through a community team	12.45
	Other (please specify) (included summary letter or medical report)	14.16
In your experience an ideal transition clinic should necessarily have… (select all that apply)	Adult neurologist	90.50
	Physiotherapist	79.75
	Occupational therapists	70.66
	Speech therapists	69.01
	Social services representatives	68.60
	Specialist nurses	68.18
	Learning disability advisors	61.57
	Dietician	52.89
	Pediatric nurses	40.50
	Other (please specify) responses included psychologists, religious officers, geneticists, or genetic counselor)	17.77
In your experience in an ideal transition clinic should necessarily include a discussion of … (select all that apply)	The young person's personal knowledge of their disease	85.54
	The young person's personal knowledge of their medications and perceived stigmatization	81.82
	Disease related co‐morbidities (such as orthopedic, gastrointestinal, or neuropsychiatric issues, risk of mortality)	78.93
	Changes in family dynamics with increasing patient age	76.03
	Sexuality, pregnancy, and reproductive issues	73.55
	General health issues (like BMI, nutrition, participation in physical activity) and well being	73.55
	Career choices and further education	72.31
	Effect of puberty on disease and medication	68.60
	Addiction, drugs, alcohol, and smoking	64.05
	Driving	59.92
	Other (please specify)	7.44

Abbreviations: BMI, body mass index; GP, general practitioner.

#### Age groups

The age by which transition from pediatric side to adult side was completed in all patients attending the service was noted as commonest for 16 to 17 (27.7%) and 17 to 18 (27.7%) year olds. There was wide variation in this age group (age range, 12–27 years). Some responders from South Asia reported on free text that adult services take on patients in their countries from 12 years. In contrast, other responders reported that transition was a prolonged process in their clinical practice and continued the care of transition patients well into their twenties.

#### Spectrum of movement disorders and treatments

Of the 148 responders offering the service, dystonia (88.5%) was the most common movement disorder seen in their service, followed by cerebral palsy (83.8%), Tourette's syndrome (70.9%), and Parkinson's disease (PD) (70.3%). Botulinum toxin injections services were provided by 41.9% and deep brain stimulation surgery was provided by 26.3% of the responders (Figure S2). In the free text, responders noted the other movement disorders covered more rarely by their transition services were ataxia (n = 3), Wilson's disease (n = 3), Huntington's disease (n = 2), myoclonus, tremors, atypical parkinsonism, neurogenetic disorders, stereotypies, drug‐induced, and functional movement disorders.

#### Transition clinic staffing

With regards to additional staffing in a transition clinic, other than the pediatric lead, an adult neurologist was noted as the commonest professional (71.6%) by the responders, followed by physiotherapists (52.7%), and speech (32.4%) and occupational therapists (29.7%). Specialist nurses were part of 21.6% of the transition clinics and another 20.9% had pediatric nurses (Table 1). When asked about staffing an ideal transition clinic, responders reported that an adult neurologist (91%) was most essential followed by a physiotherapist (80%). The majority felt the need for a specialist or pediatric nurse, speech, and occupational therapists, learning disabilities advisor, social services, and dietician (Table 1). There was also a need felt for psychologist (n = 6), religious officer (n = 3), and a geneticist (n = 3) or genetic counselor. The importance of an individualized and need‐based multidisciplinary team was also highlighted in the free text comments by five participants.

#### Issues discussed in transition clinic

Of the questions on issues discussed in transition clinics, clinical presentation, natural history, and previous and current management (69.6%) was most discussed, followed by evaluation of the young person's knowledge of disease (68.9%), comorbidities (64.2%), general health, and knowledge of medication and stigmatization. Driving was the least discussed issue (21.6%) (Table 1).

Transfer of clinical records (paper or electronic) was the commonest form of handover of medical data. Multidisciplinary reports were used by 30% (Table 1).

When asked about the ideal range of issues to be discussed within the transitional setting, responders favored discussion of all issues in the questionnaire (Appendix S1). Some respondents noted that certain aspects such as sexuality and contraception could not be discussed in the presence of several professionals and suggested that this could be better tackled in a private setting to accommodate individual cultural and religious beliefs.

#### Other pathways of transition

Of the responses, 42% had a standardized care pathway to allow transition from childhood to adulthood. In services without such a standardized transition pathway, referral to adult services (62%) was the most used method for transferring care of pediatric patients. Transfer through the general practitioner (GP) (18%) and community team (20%) were also reported (Table 1).

## Discussion

It is well recognized that transition is key to ensuring the safe and smooth transfer of patients from pediatric to adult services, particularly for complex movement disorders.^7^ There is currently very little evidence to guide movement disorders practice when it comes to transition services. In the scoping review by MDS task force,^7^ only two articles included patients with movement disorders.^10^, ^11^ This survey provides important insights into the existing transition services, namely how these are provided across the world, highlighting some key service deficiencies.

Based on responder location, transition services appear to be available across several continents and across a broad spectrum of countries with differing socio‐economic status. Overall, there was some consensus regarding transition services in terms of patient age at transition, movement disorder etiologies, staffing the service, and medical/social issues discussed.

The timing of transition should be individually assessed based on patient need and clinical situation. Although most children are thought to be ready for discussing transition by 12 years^2^ and most steps in transition can be completed by 14 to 16 years of age, there is some evidence to suggest a more individualized plan encompassing an individual's needs. In a study of 200 12 to 19 year olds using the “Am I ON TRAC for Adult Care” questionnaire, 27% of 17 year olds scored above the behavior cut‐off for transition readiness, whereas 62% 18 year olds achieved the cut‐off.^12^

**FIG. 1:**
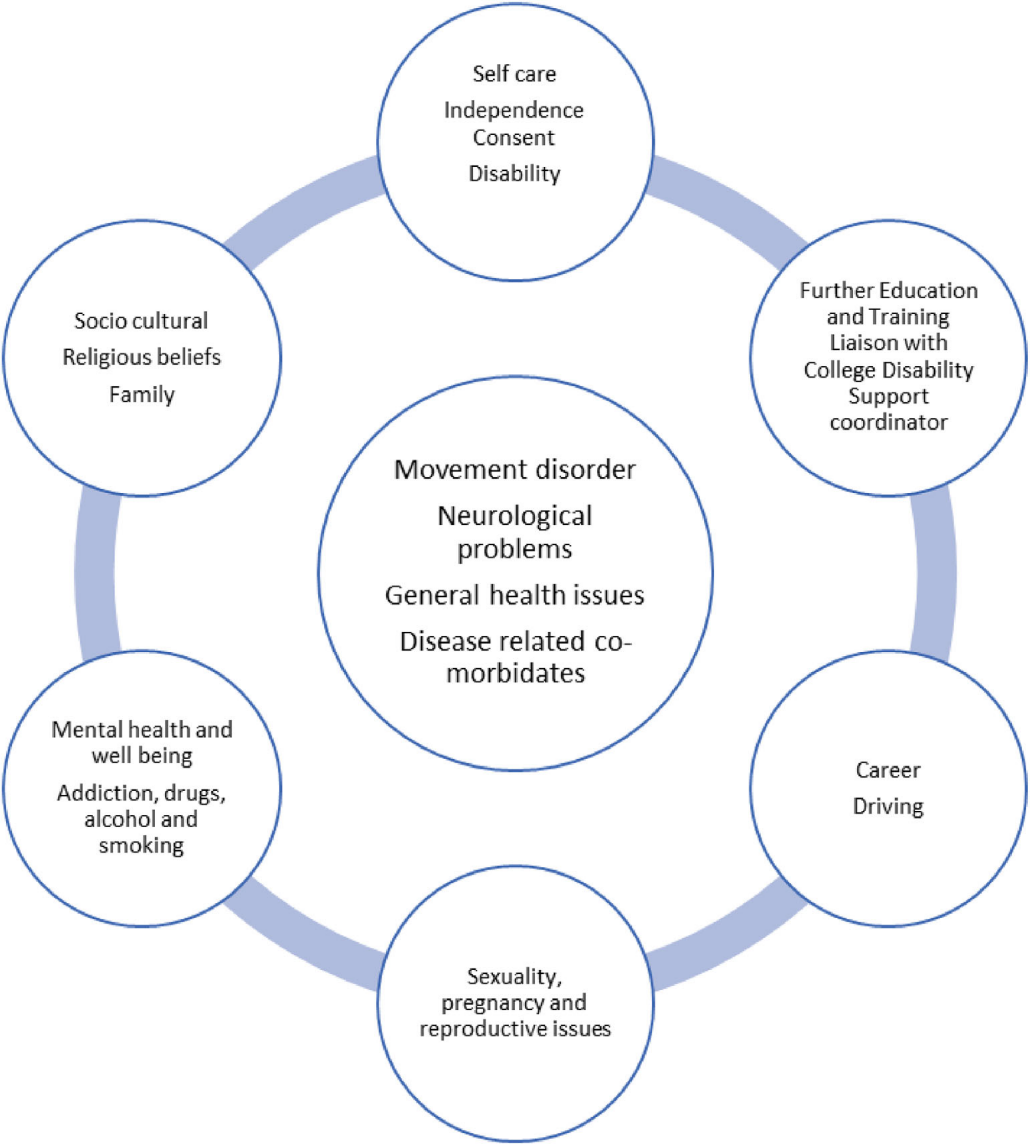
Issues discussed as part of movement disorders transition process.

In this survey, we found that some responders were offering services for a wide range of common movement disorders. We note that this survey was not designed capture the entire spectrum of the transition practice, but only the facilities available. Therefore, the results might reflect the adult neurologists' practice. For example, 70% participants offer transition for PD, which possibly reflects their ability to see PD as an adult neurology service rather than a reflection of what they encounter in a transition service. In addition to the general principles of transition planning (Figure S3), the neurological component of the transition plan forms a core part of the discussion. A joint transition clinic or overlapping follow‐up in pediatric and adult neurology can allow introduction to the adult teams and seamless continuity of care.^8^ The pre‐transition preparation should include re‐evaluation of the case history, curation of a detailed and accurate problem list including cognitive ability, transitional goals, and a transition plan that is reviewed in depth (Fig. 1). This survey did not include the frequency of attendance or waiting times. A previous study has reported that a transition clinic was provided 1 to 4 times before an adult neurologist took over full care.^8^ The waiting times for transition patients in neurology (epilepsy in United Kingdom) can vary from 1 to 12 months.^6^ It is, therefore, advisable to plan well and have an early pre‐transition visit.^5^, ^7^

In the survey, 23% of responders used a standardized pro forma or checklist. There are several models of transition checklists^13^ and toolkits available, and some are useful for neurological patients.^14^ The key principles of these tools include preparing the young person and families for independence with decision making and self‐management skills that help them achieve the full potential with education, career, driving, etc., while promoting a clear understanding of disease and prognosis.^2^, ^3^, ^4^ Moreover, inadequate preparation of patients transitioning to adult service, inconsistencies in transitional care practices, difficulty accessing adult services, and heightened emotion during the transition phase are the challenges identified with transition.^7^ Another key aspect of successful transition is the complete clinical and imaging data, and this was one of the key recommendations of a recent review,^7^ which is further substantiated by this survey. Notably, there is very little community or GP involvement in most transition services.^3^, ^6^, ^7^, ^8^ A summary of general principles of transition are presented in Figure S3.

In addition to pediatric and adult physicians, social workers, nurse specialists, and allied health professionals are usually present in transition clinics.^7^ This survey highlights that based on individualized setting a specialist or pediatric nurse, speech, and occupational therapists, learning disabilities advisor, social services, dietician, and psychologists may be an integral part of multidisciplinary transition services.

The issues that a transition service address most commonly include the adolescent's medical condition, current medications and potential side effects, signs and symptoms of concern, genetic counseling (where relevant) and reproductive implications of the condition, issues of puberty and sexuality, driving, alcohol and substance use, emotional/psychological concerns, and wellness.^3^ In this survey, when comparing issues discussed in existing services to what ideally should be discussed, the biggest gap was in the discussion of issues related to driving, addiction, alcohol, sexuality, and reproductive health issues.

Although this survey provides important insights into transition, there are several limitations. There is a reporting bias because of the low response rate and only a very small percentage of MDS members (2%) responded to the email inviting them for the survey. As per the information from the MDS secretariat, this is not an unusual response rate when using mailing lists for global surveys. This survey was not designed to capture regional differences in age of transition or conditions transitioned, such as Wilson's disease in South Asia or other rare and neurogenetic movement disorders. The survey did not include financial models, frequency, and waiting times. In future work, we plan to specifically ask about models of transitional care used and for some “good practice” tips that the respondents could share. The impact of transition care on clinical outcomes and patient satisfaction was also not studied and could be evaluated in future studies.

## Conclusion

This survey provides first‐hand data regarding existing movement disorder transition services across the globe. These results can be used to identify areas of improvement of transition care and can guide teams planning to set up transition services and transition clinics.

## Author Roles

(1) Research Project: A. Conception, B. Organization, C. Execution. (2) Statistical Analysis: A. Design, B. Execution, C. Review and Critique. (3) Manuscript: A. Writing of the First Draft, B. Review and Critique.

A.B.: 1B, 1C, 2A, 2B, 2C, 3A, 3B.

J.P.L.: 1B, 2C, 3B.

J.K.S.: 1B, 2C, 3B.

J.W.M.: 1B, 2C, 3B.

T.P.: 2C, 3B.

E.R.: 3B.

M.K.: 1A, 1B, 2B, 2C, 3B.

V.F.:1A, 1B, 1C, 2B, 3B.

## Disclosures

### Ethical Compliance Statement

The authors confirm that the approval of an institutional review board or patient consent was not required for this work because no patients were included in this study. We confirm that we have read the Journal's position on issues involved in ethical publication and affirm that this work is consistent with those guidelines.

### Funding Sources and Conflicts of Interest

The authors declare that there are no funding sources or conflicts of interest relevant to this work.

### Financial Disclosures for the Previous 12 Months

A.B. has received speaker honorarium from Ipsen Pharma and receives royalties from the book “Understanding Parkinsonism” (Jaypee brothers 2017). J.P.L. received unrestricted educational support for instructional courses and consultancy fees from Medtronic. J.K.S. reports no disclosures. J.W.M. has been funded by the National Institutes for Health (NIH). He has received compensation as a consultant for Biogen, Applied Therapeutics, Neurogene, Taysha Gene Therapies, and Amicus Therapeutics. He serves on DSMBs for PTC Therapeutics. He has received research support through the University of Rochester from Beyond Batten Disease Foundation, Abeona Therapeutics, Amicus Therapeutics, and Neurogene. T.P. receives salary support from the American Academy of Neurology and holds research grants from the Maternal Newborn Child and Youth Strategic Clinical Network, the Owerko Centre of Alberta Children's Hospital Research Institute, and the Canadian Institutes of Health Research. E.R. received honorarium for speech from Orkyn, Aguettant, and Elivie, and served on advisory boards from Allergan. He received research support from Merz‐Pharma, Orkyn, Aguettant, Elivie, Ipsen, Allergan, Everpharma, Fondation Desmarest, AMADYS, ADCY5.org, Agence Nationale de la Recherche, Societé Française de Médecine Esthétique, and Dystonia Medical Research Foundation. M.K. is a NIH Research Professor and recipient of the Sir Jules Thorn Award for Biomedical Research, with additional research funding from the RoseTrees Trust and Great Ormond Street Hospital Children's Charity. V.F. receives a salary from New South Wales Health; has received unrestricted research grants from The Michael J. Fox Foundation, AbbVie, and Merz; is on Advisory Boards and/or has received travel grants from AbbVie, Allergan, Cavion, Ipsen, Merz, Praxis, Seqirus, Stada, Teva, and UCB; and receives royalties from HealtPress.

## Supporting information


**Figure S1.** Map of countries of practice of the respondents to the survey.

**Figure S2.** Conditions seen in transition clinics.

**Figure S3.** General principles of transition.

**Appendix S1.** MDS Task Force on Pediatrics—transition services for children survey.

**Appendix S2.** Additional information—details of the survey and analysis.
