# Supplementary figures and images for: Transition Services for Children and Young Adults with Movement Disorders: A Survey by the MDS Task Force on Pediatrics
Source: Mov Disord Clin Pract. 2022 Sep 28;9(7):972–8. doi: 10.1002/mdc3.13549 (PMC9547133; doi:10.1002/mdc3.13549)

Map of the countries of respondents who participated in the MDS task force transition survey

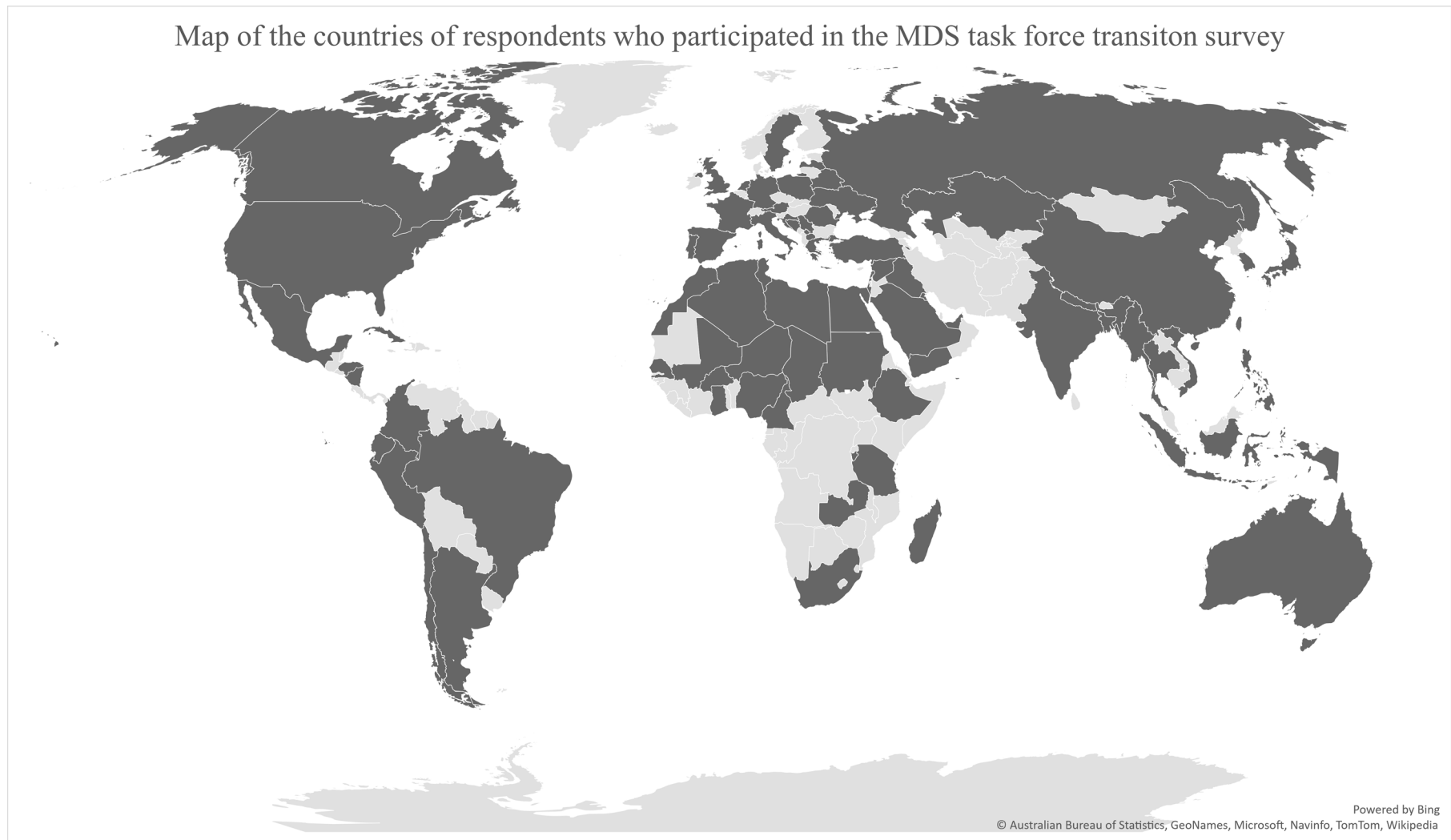

Supplement: Supplementary file 1 — Figure S1. Map of countries of practice of the respondents to the survey. [file MDC3-9-972-s001.pdf]

Supplemental figure 2. Conditions seen in transition clinics and services provided

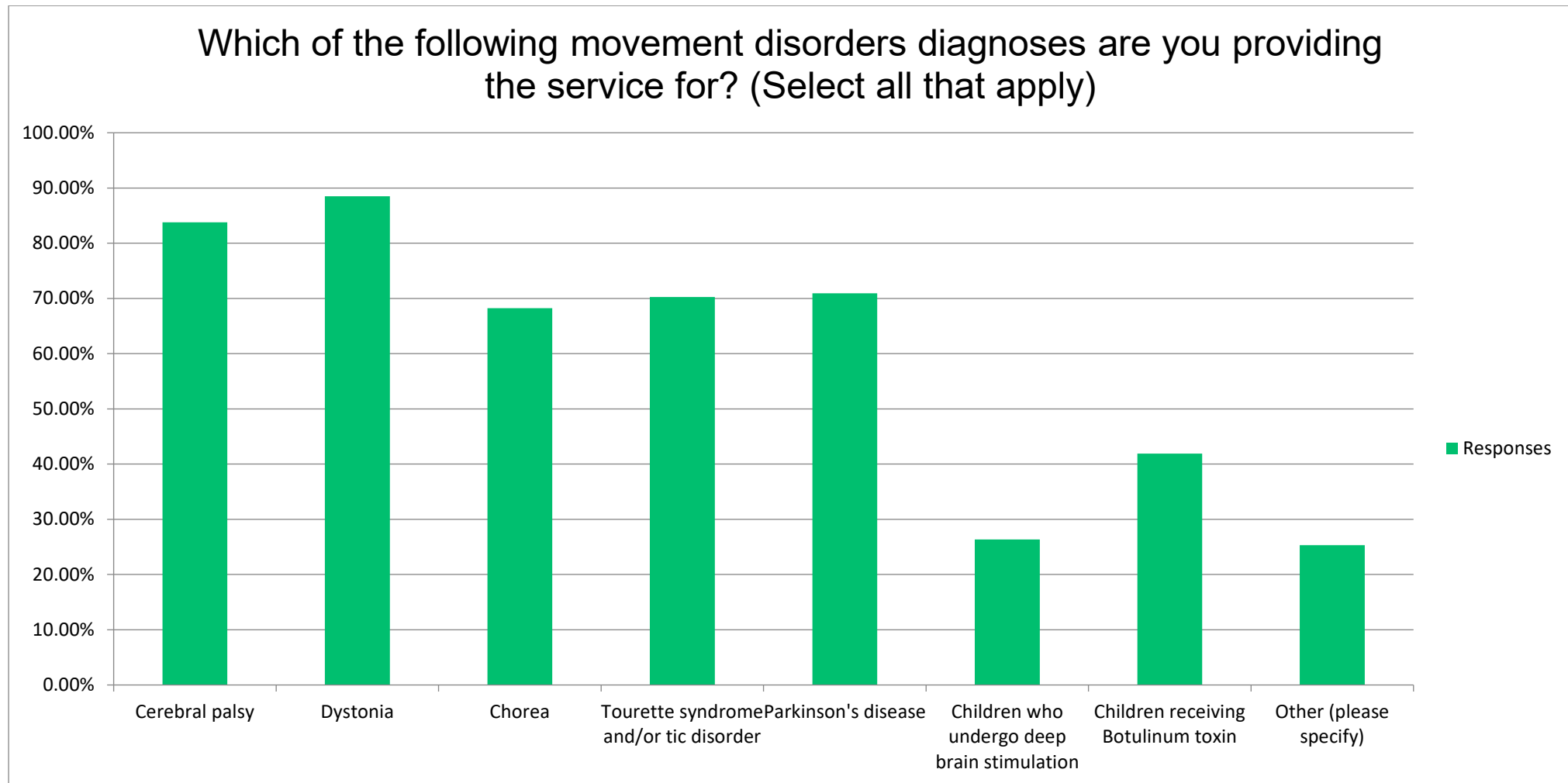

Supplement: Supplementary file 2 — Figure S2. Conditions seen in transition clinics. [file MDC3-9-972-s002.pdf]
