# Supplementary material for: Transition Services for Children and Young Adults with Movement Disorders: A Survey by the MDS Task Force on Pediatrics
Source: Mov Disord Clin Pract. 2022 Sep 28;9(7):972–8. doi: 10.1002/mdc3.13549 (PMC9547133; doi:10.1002/mdc3.13549)
Supplement: Supplementary file 3 — Figure S3. General principles of transition. [file MDC3-9-972-s004.pdf]

**General principles of transition service**

1. Initial planning visit to discuss transition with the patient and family (around age 12-14)
2. Pre transition preparation to include case history, problem list, transitional goals, and a transition plan
3. Multidisciplinary approach (pediatric and adult neurologist with nurses and therapists) approach to cover broad transition issues
4. Joint transition clinic or overlapping follow-up in pediatric and adult neurology to allow adult neurologist to introduce and take over care (1-4 visits over 1-4 years)
