# Supplementary material for: Transition Services for Children and Young Adults with Movement Disorders: A Survey by the MDS Task Force on Pediatrics
Source: Mov Disord Clin Pract. 2022 Sep 28;9(7):972–8. doi: 10.1002/mdc3.13549 (PMC9547133; doi:10.1002/mdc3.13549)
Supplement: Supplementary file 4 — Appendix S1. MDS Task Force on Pediatrics—transition services for children survey. [file MDC3-9-972-s003.pdf]

SurveyMonkey

Is this the survey you're looking for? Try creating your own with the world's leading platform.

SIGN UP FREE LEARN MORE

MDS Task Force on Pediatrics - Transition Services for Children with Neurological Disorders

1. Name:

2. Department:

3. Hospital:

4. City:

5. County/State:

6. Country:

SurveyMonkey

Is this the survey you're looking for? Try creating your own with the world's leading platform.

SIGN UP FREE LEARN MORE

with movement disorders in your service?

- Yes
- No

About your service:

8. What is the youngest age of patients attending your service?

- 12
- 13
- 14
- 15
- Other (please specify)

9. By what age is the transition from paediatric side to adult side complete in all patients attending your service

- 16-17
- 17-18
- 18-19
- 19-21
- Other (please specify)

SurveyMonkey

Is this the survey you're looking for? Try creating your own with the world's leading platform.

SIGN UP FREE LEARN MORE

(Select all that apply)

- [ ] Cerebral palsy
- [ ] Dystonia
- [ ] Chorea
- [ ] Tourette syndrome and/or tic disorder
- [ ] Parkinson's disease
- [ ] Children who undergo deep brain stimulation
- [ ] Children receiving Botulinum toxin
- [ ] Other (please specify)

11. In addition to a Pediatric consultant/Doctor, who all are present in the transition clinic at your service? (Select all that apply)

- [ ] Adult Neurologist
- [ ] Specialist nurses
- [ ] Learning disability advisors
- [ ] Physiotherapist
- [ ] Occupational therapists
- [ ] Speech therapists
- [ ] Pediatric nurses
- [ ] Social services representatives
- [ ] Other (please specify)

12. What issues are discussed in the transition service? (Select all that apply)

SurveyMonkey

Is this the survey you're looking for? Try creating your own with the world's leading platform.

SIGN UP FREE LEARN MORE

- [ ] The young person's personal knowledge of their medications and perceived stigmatisation
- [ ] Effect of puberty on disease and medication
- [ ] Sexuality, pregnancy and reproductive issues
- [ ] Addiction, drugs, alcohol and smoking
- [ ] Changes in family dynamics with increasing patient age
- [ ] Other (please specify)
- [ ] Driving
- [ ] General health issues (like BMI, nutrition, participation in physical activity) and well being
- [ ] Disease related co-morbidities (such as orthopedic, gastrointestinal or neuropsychiatric issues, risk of mortality)
- [ ] Clinical presentation, natural history and previous and current management

13. How is the clinical data transferred from pediatric to transitional/adult services? (Select all that apply)

- [ ] Standardized proformas
- [ ] Transfer of clinical records

SurveyMonkey

Is this the survey you're looking for? Try creating your own with the world's leading platform.

SIGN UP FREE LEARN MORE

14. What other ways or options are available to you to allow safe transition from childhood to adulthood for your patients with movement disorders? (Select all that apply)

- [ ] Referral services to adult clinics within your hospital/service
- [ ] Transfer of clinical records and/or neuroimaging through a standardized care pathway
- [ ] Transfer of clinical records and/or neuroimaging through GP
- [ ] Transfer of clinical records and/or neuroimaging through a community team
- [ ] Other (please specify)

15. In your experience an ideal transition clinic should necessarily have? (Select all that apply)

- [ ] Adult Neurologist
- [ ] Speech therapists
- [ ] Specialist nurses
- [ ] Pediatric nurses
- [ ] Learning disability advisors
- [ ] Social services representatives

SurveyMonkey

Is this the survey you're looking for? Try creating your own with the world's leading platform.

SIGN UP FREE LEARN MORE

- [ ] Other (please specify)

16. In your experience in an ideal transition clinic should necessarily include a discussion of? (Select all that apply)

- [ ] The young person's personal knowledge of their disease
- [ ] The young person's personal knowledge of their medications and perceived stigmatisation
- [ ] Effect of puberty on disease and medication
- [ ] Sexuality, pregnancy and reproductive issues
- [ ] Addiction, drugs, alcohol and smoking
- [ ] Changes in family dynamics with increasing patient age
- [ ] Career choices and further education
- [ ] Driving
- [ ] General health issues (like BMI, nutrition, participation in physical activity) and well being
- [ ] Disease related co-morbidates (such as orthopedic, gastrointestinal or neuropsychiatric issues, risk of mortality)
- [ ] Other (please specify)

SurveyMonkey

Is this the survey you're looking for? Try creating your own with the world's leading platform.

SIGN UP FREE LEARN MORE

Done

Powered by

SurveyMonkey

See how easy it is to create a survey.

Privacy & Cookie Policy
