# Supplementary material for: Transition Services for Children and Young Adults with Movement Disorders: A Survey by the MDS Task Force on Pediatrics
Source: Mov Disord Clin Pract. 2022 Sep 28;9(7):972–8. doi: 10.1002/mdc3.13549 (PMC9547133; doi:10.1002/mdc3.13549)
Supplement: Supplementary file 5 — Appendix S2. Additional information—details of the survey and analysis. [file MDC3-9-972-s005.docx]

Appendix 2 Additional information -**Details of the survey and analysis**

The questions were designed to capture a number of key transition issues, including (i) location of transition services (ii) patient age at review in transition clinic (iii) issues discussed in transition clinic and (iv) alternative ways of providing transition support services where clinics do not currently exist. The questionnaire was digitalized as an online survey (using SurveyMonkey®) and sent to all members between October 2020 and June 2021. Administrative support from the MDS was used to disseminate the online survey to all members of the MDS. The data was collated using Microsoft Excel. The data was screened for blank responses, errors, and duplicates. The data was standardized to remove mix of numbers and word. For analysis as the response number was above 50, we decided to use percentage of responses to measure proportions and scale of the responses. Free text comments were reviewed and analyzed to include relevant data. The critical analysis included a review of themes, trends and patterns and identify any gaps. We used Revised Standards for Quality Improvement Reporting Excellence (SQUIRE 2.0) for reporting the survey results as a quality improvement project.

**Geographical location of participants**

The largest group of responders were from the USA (n=29). Ten or more responses were received from India, Egypt, Canada, Nigeria, Brazil, China, Germany, Argentina, Italy, UK, Romania, Spain and five or more responses from Japan, Indonesia, Philippines, Mexico, Peru, Cameroon, and France.
